# Prediction of absenteeism in public schools teachers with machine learning

**DOI:** 10.11606/s1518-8787.2021055002677

**Published:** 2021-06-07

**Authors:** Fernando Timoteo Fernandes, Alexandre Dias Porto Chiavegatto

**Affiliations:** I Universidade de São Paulo Faculdade de Saúde Pública Programa de Pós-Graduação em Saúde Pública São PauloSP Brasil Universidade de São Paulo. Faculdade de Saúde Pública. Programa de Pós-Graduação em Saúde Pública. São Paulo, SP, Brasil; II Fundacentro São PauloSP Brasil Fundacentro. São Paulo, SP, Brasil; III Universidade de São Paulo Faculdade de Saúde Pública São PauloSP Brasil Universidade de São Paulo. Faculdade de Saúde Pública. São Paulo, SP, Brasil

**Keywords:** Absenteeism, Risk Factors, Supervised Machine Learning, School Teachers, Early Childhood Education

## Abstract

**OBJECTIVE:**

To predict the risk of absence from work due to morbidities of teachers working in early childhood education in the municipal public schools, using machine learning algorithms.

**METHODS:**

This is a cross-sectional study using secondary, public and anonymous data from the *Relação Anual de Informações Sociais*, selecting early childhood education teachers who worked in the municipal public schools of the state of São Paulo between 2014 and 2018 (n = 174,294). Data on the average number of students per class and number of inhabitants in the municipality were also linked. The data were separated into training and testing, using records from 2014 to 2016 (n = 103,357) to train five predictive models, and data from 2017 to 2018 (n = 70,937) to test their performance in new data. The predictive performance of the algorithms was evaluated using the value of the area under the ROC curve (AUROC).

**RESULTS:**

All five algorithms tested showed an area under the curve above 0.76. The algorithm with the best predictive performance (artificial neural networks) achieved 0.79 of area under the curve, with accuracy of 71.52%, sensitivity of 72.86%, specificity of 70.52%, and kappa of 0.427 in the test data.

**CONCLUSION:**

It is possible to predict cases of sickness absence in teachers of public schools with machine learning using public data. The best algorithm showed a better result of the area under the curve when compared with the reference model (logistic regression). The algorithms can contribute to more assertive predictions in the public health and worker health areas, allowing to monitor and help prevent the absence of these workers due to morbidity.

## INTRODUCTION

Professionals in the area of basic education have high rates of sickness absence due to work-related illness, ranking among the first positions in Brazil^1^. Among its main diseases are voice disorders, diseases of the respiratory and musculoskeletal system, as well as mental and behavioral disorders^2,3^. Notifications related to mental health have recently increased, which is the case of the state of São Paulo^4^. The working conditions to which these professionals are exposed, with long work days, large classes^5^ and lack of recognition, tend to increase the problem, with direct consequences in the private lives of teachers^6^.

However, there are still few indicators that analyze the conditions of teachers at each stage of basic education, from early childhood education to high school, areas that show distinctions and specificities in the work context^7^. Studies that seek to generate indicators usually use aggregate values of the different stages of basic education^1^ or are based on surveys in specific regions that depend on the action of the respondent, and can generate high operational costs^8,9^.

The use of aggregate indicators for the analysis of diseases can omit critical situations of morbidity and do not correspond to the reality of the different working conditions to which teachers are exposed. The problem is aggravated by analyzing teachers from the municipal public schools, whose sick leave data are only accessible through health departments or medical expertise departments of each municipality^2^. One way to analyze the morbidity situation by teaching stage and the relations of work with teachers’ illness is to look for official public data sources, which have individualized and updated records with wide geographical coverage.

In this scenario, from 2011, the publication of Law No. 12,527 of 2011, known as *Lei de Acesso à Informação* (LAI – Access to Information Law) has expanded access to data from the federal government. This legislation allows information to be requested from any public body and provides for public entities and bodies to anticipate and publish their data and information on the internet in open and non-proprietary formats, known as open data^10^. An example is the *Relação Anual de Informações Sociais* (RAIS – Annual Social Information Report), instituted by Decree No. 76,900 of December 23, 1975, currently under the responsibility of the Ministry of Economy. Data are disclosed annually in a public and anonymous way, containing individualized records with coverage throughout the national territory^11^.

The main purpose of RAIS is to subsidize the generation of labor statistics and the formal market (private and statutory) for government entities. However, types of leave are also available, such as illness or work-related illness, without information of the specific disease. Filling is mandatory for all public and private companies since 1977^12^. In the data on teacher sick leave in RAIS, we found that, on average, more than 40% of teachers in early childhood education were absent from work at least once a year due to morbidities between 2014 and 2018, surpassing the other stages of basic education and pointing to the need to analyze this specific group.

This study proposes to build predictive models capable of estimating the risk of absence from work of early childhood education teachers working in the municipal public schools, considering all municipalities in the state of São Paulo, using artificial intelligence and supervised learning algorithms (machine learning) known for high performance, especially in the health area^13^. We expect that the best proposed model may be applied to estimate the number of sick leave in teachers, using projected data from workers (e.g. time of occupation, workload and income) and the work environment (e.g. students per class), in order to subsidize targeted public policies to prevent absence from work, avoiding new illnesses, improving the quality of life of these professionals and, consequently, the quality of education in the public schools.

## METHODS

This is a cross-sectional study to predict the risk of absence from work due to morbidity (disease or work-related disease) in teachers working in early childhood education (preschool period) of the entire municipal public network of the state of São Paulo, by using secondary, public and anonymous data.

### Data Sources

We used Individualized data at the lowest level of disaggregation, also known as microdata^11^, of formal employment links provided by RAIS in the period between 2014 and 2018. The RAIS maintains information provided annually by public and private companies on the formal employment relationships of its employees, such as hires and dismissals made in the current year, as well as data on workday and leave due to illness or work-related illness. This study included teachers from the municipal public schools of all municipalities in the state of São Paulo who reported data to RAIS, in which teachers had a higher level and active employment relationship until December 31 of the year working in preschool education, classified in RAIS with CBO 231105 (Higher Education Teachers in Children’s Education, four to six years).

As a form of identification of the outcome, we used the fields of cause of absence in the RAIS, filtering by codes 30 (occupational disease) and 40 (disease). Retirements due to illness were not considered. To identify the type of establishment (federal, state, municipal or private), we used information on the legal nature of the establishment (codes 1031, 1066, 1120, 1155, 1180 and 1244). To identify the activity of the establishment, code 841160 (general public administration) and division 85 (education) of the National Classification of Economic Activities version 2.0 (CNAE 2.0) provided in RAIS were used.

About 99.7% (n = 174,346) of the records were registered in CNAE 841160, and the rest, 0.03% (n = 52), belonged to a CNAE not related to preschool education, which was thus excluded from the selection. We also excluded records in which the municipality of employment of the worker did not correspond to the Federal unit of São Paulo, resulting in a total of 174,294 records. In situations in which the municipality of employment of the worker was not informed, the municipality of the establishment was adopted.

To understand part of the working conditions that teachers were exposed to, we linked the information on the average number of students per classroom in early childhood education, provided by the National Institute for Educational Studies and Research Anísio Teixeira (INEP)^14^, considering that some of the diseases common to all faculty members are associated with the acoustic environment and with the complexity of the activities of the teacher^3^, and added it to the number of inhabitants for the municipality, which is provided by the Fundação Seade^15^. The data from INEP and Fundação Seade were linked to the selected sample of RAIS through the municipality code of the Brazilian Institute of Geography and Statistics (IBGE).

A total of 11 predictor variables were selected: sex, age, post-graduation, size of the establishment, type of employment relationship, first employment, time in employment, hours hired, annual average of the amount of minimum wages, average of students per class and number of inhabitants in the municipality where the teacher worked.

### Data Preparation

The data was preprocessed to check missing values and transform variables, so they could be used to build different types of predictive machine learning models^16^. All selected variables were fully filled, with no missing values. Variables with more than two categories were represented by a set of variables called dummy, in which for each category, a new variable with values of 0 or 1 is generated. Continuous variables were standardized with z-score. After testing the numerical variables, we found a high correlation between the number of inhabitants in the municipality and the average number of students (0.93); thus, we chose to dichotomize the number of inhabitants variable, using the definition of 500 thousand inhabitants to identify performance in a large city^17^.

For the preprocessing and data loading phase, we used the MS SQL Server Database Manager system. To analyze the data and build the predictive models, we used the R software.

### Construction of predictive models

In all, five supervised machine learning algorithms were developed: logistic regression, decision trees^16^, random forest^18^, XGBoost^19^ and artificial neural networks^20^. Machine learning algorithms are subject to problems such as underfitting – when the model cannot adjust to the inherent variability of the data, generating poor estimates – and overfitting – when the model fits very well for the sample used in the training, but does not get good results for new data. One way to improve the performance of algorithms and avoid problems such as overfitting or underfitting is to apply resampling and cross-validation techniques for the selection of hyperparameters^16^.

In cross-validation, the dataset is splitted into two parts. The first part is intended for training the algorithm, and the second is used to adjust the hyperparameters of the model, simulating new data and selecting hyperparameters that optimize the chosen performance metric. This training phase allows the algorithm to achieve better predictive performance when new data sets are presented. One of the most well-known cross-validation techniques is k-fold, in which *k* means the number of subsets of test data that will be used for hyperparameter adjustments of the model during the training phase^21^.

For model development, a part of the data (60%) has been allocated for training using the period between 2014 and 2016 (n = 103,357), including cross-validation with the k-fold technique using 10 partitions for definition of hyperparameters. The other part of the records (40%), referring to the period between 2017 and 2018 (n = 70,937), was used to evaluate the performance of the models, omitting the result on sick leave and applying the trained model to estimate the outcome in future data. To evaluate the performance of the models, measures such as accuracy, sensitivity, specificity, positive predictive value (PPV) and negative predictive value (NPV) were analyzed. To select the best model, we used the value of the area under the receiver operating characteristic curve (AUROC)^22^. Cohen’s kappa coefficient was used to evaluate the agreement between the predicted and observed values.

This study follows the guidelines for the description of multivariate predictive models for diagnosis or prognosis (Transparent Reporting of a Multivariable Prediction Model for Individual Prognosis or Diagnosis – TRIPOD)^23^.

### Confidentiality criteria

The project was approved by the Research Ethics Committee of the Faculdade de Saúde Pública of the Universidade de São Paulo, under no. 4.031.362, CAAE 30786620.5.0000.5421.

## RESULTS

### Study Population

Initially, we conducted the descriptive analysis of training (2014 to 2016) and test data (2017 to 2018). The training data comprise 103,357 records of teachers’ employment relationships, with 45,419 (43.94%) records of sick leave or work-related illness. The test data correspond to 70,937 records and 30,261 cases of sick leave (42.65%). Table 1 shows the results of this analysis.

**Table 1:** Descriptive analysis of training data (n = 103,357) and test data (n = 70,937).

Variable	Category	Training		Test	
		n	%	n	%
Sex	0 – Male	22,120	21.40	16,055	22.63
	1 – Female	81,237	78.60	54,882	77.37
Post-graduation	0 – No	100,904	97.63	68,412	96.44
	1 – Yes	2,453	2.37	2,525	3.56
Age^a^	-	43.74 (9.73)		43.97 (9.70)	
Large company^b^	0 – No	19	0.02	19	0.03
	1 – Yes	103,338	99.98	70,918	99.97
Type of relationship	0 – Statutory	96,682	93.54	65,210	91.93
	1 – CLT (Brazilian Consolidation of Labor Laws)	5,867	5.68	4,493	6.33
	2 – Temporary	808	0.78	1,234	1.74
First job	0 – No	100,105	96.85	65,236	91.96
	1 – Yes	3,252	3.15	5,701	8.04
Time in employment (months)^a^	-	120.49 (92.31)		123.29 (93,84)	
Hours hired^a^	-	33.28 (6.60)		33.28 (6.33)	
Minimum wages^a^	-	5.03 (3.04)		5.04 (3.04)	
Large city^c^	0 – up to 500 thousand inhab.	30,866	29.86	22,212	31.31
	1 – above 500 thousand inhab.	72,491	70.14	48,725	68.69
Students per class^a^	-	27.42 (5.75)		26.74 (5.50)	

Inhab.: Inhabitants

^a^ Mean (standard deviation).

^b^ Above 100 employees.

^c^ Classification of cities by the Organização para a Cooperação e Desenvolvimento Econômico (OCDE – Organization for Economic Cooperation and Development)^17^: small, medium, large (over 500 thousand inhabitants) and metropolis.

Then, we conducted the analysis of independent variables to verify whether there is a significant relation with the outcome, using the Pearson Chi-square association test for categorical variables. Table 2 shows the results and allows us to observe that most variables have a significant association with the outcome.

**Table 2:** Analysis of association with the outcome of the training sample.

Variable	Category	Teachers in sick leave			Test c^2a^
		Yes	No	Total	
		n (%)	n (%)	n (%)	
Worker data					
Sex	0 – Male	9,930 (44.89)	12,190 (55.11)	22,120 (100.0)	< 0.001
	1 – Female	35,489(43.69)	45,748 (56.31)	81,237 (100.0)	
Post-graduation	0 – No	45,075 (44.67)	55,829 (55.33)	100,904 (100.0)	< 0.0001
	1 – Yes	344 (14.02)	2,109 (85.98)	2,453 (100.0)	
Age	43.74 (9.73)				
Company data					
Large company^b^	0 – No	11 (57.89)	8 (42.11)	19 (100.00)	0.220
	1 – Yes	45,408 (43.94)	57,930 (56.06)	103,338 (100.00)	
Employment data					
Type of relationship	0 – CLT (Brazilian Consolidation of Labor Laws)	746 (12.72)	5,121 (87.28)	5,867 (100.0)	< 0.0001
	1 – Statutory	44,500 (46.03)	52,182 (53.97)	96,682 (100.0)	
	2 – Temporary	173 (21.41)	635 (78.59)	808 (100.0)	
First job	0 – No	44,616 (44.57)	55,489 (55.43)	100,105 (100.0)	< 0.0001
	1 – Yes	803 (24.69)	2.449 (75.31)	3,252 (100.0)	
Time in employment (months)	120.49 (92.31)				
Hired hours	33.28 (6.60)				
Minimum wages	5.03 (3.04)				
Environmental data					
Large city^c^	0 – up to 500 thousand inhab.	3,422 (11.09)	27,444 (88.91)	30,866 (100.0)	< 0.0001
	1 – above 500 thousand inhab.	41,997 (57.93)	30,494 (42.07)	72,491 (100.0)	
Students per class	27.04 (5.60)				

Inhab.: Inhabitants.

^a^ Association test by c^2^: *H*_0_: no association; H_a_: there is association.

^b^ Above 100 employees.

^c^ Classification of cities by the Organização para a Cooperação e Desenvolvimento Econômico (OCDE – Organization for Economic Cooperation and Development)^17^: small, medium, large (over 500 thousand inhabitants) and metropolis.

Note: Mean values (standard deviation).

### Analysis of Predictive Models

**Figure 1:**
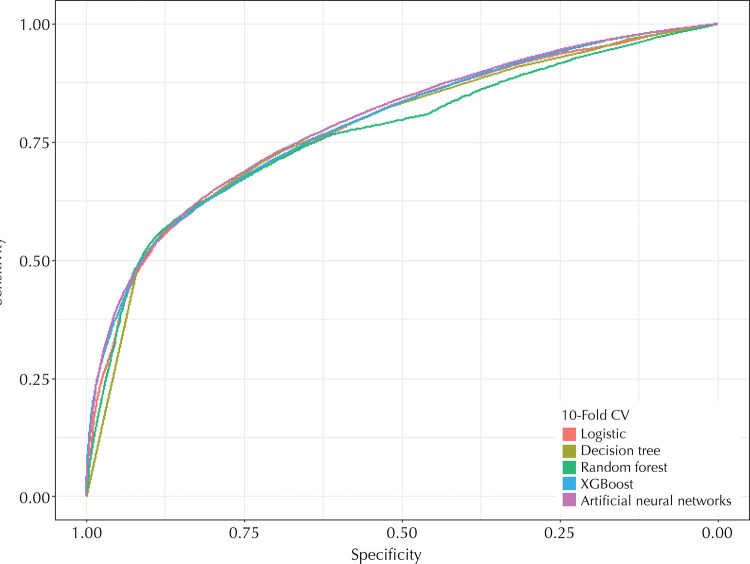
Comparison of models based on prediction results using test data (2017 and 2018).

Figure 1 shows the ROC curves of each developed model, in which 11 variables were included (type of employment relationship, number of hours hired, employment time in months, average amount of minimum wages in a year, sex, size of the establishment, post-graduation, age, first job, average of students per preschool class in the municipality and number of inhabitants).

Table 3 shows the performance results for each algorithm. Artificial neural networks achieved best performance with 0.79 area under the curve, 71.52% accuracy, 72.86% sensitivity, 70.52% specificity, PPV of 64.77% and NPV of 77.74%. The model also resulted in the best kappa coefficient, of 0.4266.

**Table 3:** Results of models developed using test data.

Algorithm	AUC	Sensitivity	Specificity	Accuracy	PPV	NPV	Kappa
Logistic regression	0.7792 [0.7759–0.7826]	0.7754	0.6568	0.7074	0.6270	0.7972	0.4195
Decision tree	0.7756 [0.7722–0.7790]	0.7008	0.7243	0.7142	0.6541	0.7649	0.4212
Random forest	0.7670 [0.7634–0.7703]	0.7715	0.6571	0.7059	0.6260	0.7944	0.4134
XGBoost	0.7843 [0.7810–0.7876]	0.7235	0.6970	0.7083	0.6398	0.6970	0.4136
Artificial neural networks	0.7902 [0.7867–0.7934]	0.7286	0.7052	0.7152	0.6477	0.7774	0.4266

AUC: area under the curve.

The hyperparameters of the machine learning models that achieved the highest AUC are as follows: decision tree (cp-complexity parameter = 0.000385), random forest (mtry = 7), XGBoost (nrounds = 100, max_depth = 4, gamma = 0.6, colsample_bytree = 0.8, min_child_weight = 2, subsample = 1), and artificial neural networks (size = 19, decay = 0.2).

Each algorithm uses a different set of rules to fit the data with the least possible error, and some variables become more important than others for the predictive ability of the algorithm. The importance of the variables refers to how much a variable contributed to making accurate predictions in each model. For linear models, traditional t statistics are used to account for the importance of variables. In the other models, the importance depends on the implementation of each algorithm.

For example, in the case of decision trees, if a variable is used more frequently in leaf divisions, contributing to higher node purity, it will be of greater importance to the final model, meaning that its removal will impair the overall performance of the model. In models that use multiple trees, such as random forest, the importance of the variables is calculated at each division of the leaves of a tree and summed with the result of the other trees^21^. In neural network models, this study used the weights^24^ method, in which a combination of the output values of the neurons of the hidden layers and the values of the weights of each connection was used to estimate the greatest contribution and representativeness of each input variable in the classification result. In this study, the R caret package^25^ was applied, which already implements the estimation of importance of variables according to each model^26^.

**Figure 2:**
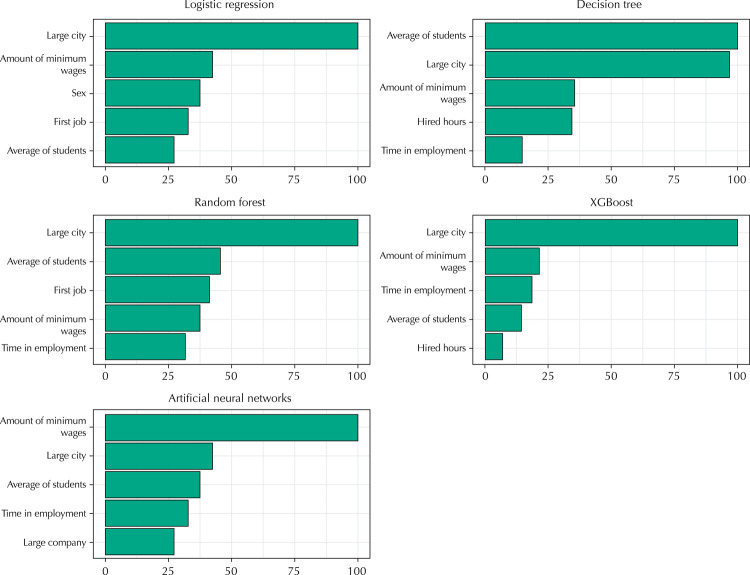
Importance of variables by predictive model.

Although they do not have causal interpretation^27^, the variables can give relevant information about their relation with the outcome. Figure 2 shows how each variable contributed to the models developed.

The variables that most contributed to the best model (artificial neural networks) were: amount of minimum wages, large city, average of students per class, time in employment and large company.

## DISCUSSION

The RAIS can provide indications of the morbidity situation of workers by means of the field of cause of sick leave. The wide coverage and individualized registration facilitate data selection and analysis from a population of formal workers with the same occupation in a given state, which allows to monitor, over the years, the evolution of sick leave and predict which professionals have the highest risk of illness in the coming years. For the best algorithm (artificial neural networks), we obtained an area under the ROC curve of 0.79, 72.86% sensitivity and 71.52% accuracy, using only 11 predictor variables.

The results show the feasibility of using anonymized sources to predict the morbidity of preschool teachers with machine learning algorithms. The variables that contributed the most to the best model can be explored in future studies to verify if there is a causal relation. The size of the city in which one works, average income of the worker and average of students per class may have direct relation on the health of teachers. In the case of the average of students per class, there is evidence in literature that corroborates this reasoning, such as problems in the acoustics of the environment and high noise due to greater agitation in the classroom^3,28^.

Machine learning algorithms have been used in different contexts in worker health to help predict morbidities related to the activities of these professionals^29,30^. In the area of education, no studies used machine learning or other predictive models for the risk of teacher absenteeism as the main outcome, while some studies analyzed the association of predictor variables with the outcome^31,32^, but its prediction was not the main objective. The prediction of teacher absenteeism can identify teachers who need greater attention, allowing to adopt measures to avoid sick leave due to diseases related to the occupation^3,28^, reducing absence from work and consequently improving the quality of teaching^33^.

The study has some limitations. Since we used anonymous data, it was not possible to verify whether the teachers, who may have multiple municipal employment ties, work in more than one school. It was also not possible to identify whether there was a previous absence due to a pre-existing disease that may be related to subsequent sick leaves. Finally, a low number of predictor variables was analyzed due to the availability of RAIS. We expect that, in the future, with a more extensive collection conducted with questionnaires, even more robust results will be possible on the part of the algorithms.

In conclusion, only with public and anonymous data, it was possible to obtain good sensitivity (73%) and specificity (71%) in identifying the risk of absenteeism due to illness, regardless of the cause of morbidity. Although it was not created for health purposes, the RAIS proved to be feasible for the analysis of absenteeism due to morbidities, mainly because it contains variables of employment relationships, and can be explored in other professional categories. The developed models can be applied in the future in other teaching stages and in other occupational categories. Machine learning algorithms can contribute to more assertive predictions in the analysis of absenteeism of early childhood teachers in the municipal public network, and may be used in the development of indicators of morbidity or to subsidize preventive public policies aimed at this professional category.
