# Supplementary material for: Prediction of absenteeism in public schools teachers with machine learning
Source: Rev Saude Publica. 2021 Jun 7;55:23. doi: 10.11606/s1518-8787.2021055002677 (PMC8225323; doi:10.11606/s1518-8787.2021055002677)
Supplement: Translation

# Translation: Predição de absenteísmo docente na rede pública com machine learning

**Authors:** Fernando Timoteo Fernandes, Alexandre Dias Porto Chiavegatto

**Affiliations:** I Brasil Universidade de São Paulo. Faculdade de Saúde Pública. Programa de Pós-Graduação em Saúde Pública. São Paulo, SP, Brasil; II Brasil Fundacentro. São Paulo, SP, Brasil; III Brasil Universidade de São Paulo. Faculdade de Saúde Pública. São Paulo, SP, Brasil

**Keywords:** Absenteísmo, Fatores de Risco, Aprendizado de Máquina Supervisionado, Professores Escolares, Educação Infantil

## Abstract

**OBJETIVO:**

Predizer o risco de ausência laboral decorrente de morbidades dos docentes que atuam na educação infantil na rede pública municipal, com o uso de algoritmos de *machine learning*.

**MÉTODOS:**

Trata-se de um estudo transversal utilizando dados secundários, públicos e anônimos da Relação Anual de Informações Sociais, selecionando professores da educação infantil que atuaram na rede pública municipal do estado de São Paulo entre 2014 e 2018 (n = 174.294). Foram também vinculados dados da média de alunos por turma e número de habitantes no município. Os dados foram separados em treinamento e teste, utilizando os registros de 2014 a 2016 (n = 103.357) para treinar cinco modelos preditivos e os dados de 2017 a 2018 (n = 70.937) para testar seus desempenhos em dados novos. A performance preditiva dos algoritmos foi avaliada por meio do valor da área abaixo da curva ROC (AUROC).

**RESULTADOS:**

Todos os cinco algoritmos testados apresentaram área abaixo da curva acima de 0,76. O algoritmo com melhor performance preditiva (redes neurais artificiais) obteve 0,79 de área abaixo da curva, com acurácia de 71,52%, sensibilidade de 72,86%, especificidade de 70,52% e kappa de 0,427 nos dados de teste.

**CONCLUSÃO:**

É possível predizer casos de afastamentos por morbidade em docentes da rede pública com *machine learning* usando dados públicos. O melhor algoritmo apresentou melhor resultado da área abaixo da curva quando comparado ao modelo de referência (regressão logística). Os algoritmos podem contribuir para predições mais assertivas na área da saúde pública e da saúde do trabalhador, permitindo acompanhar e ajudar a prevenir afastamentos por morbidade desses trabalhadores.

## INTRODUÇÃO

Profissionais da área da educação básica apresentam altos índices de afastamento por doença relacionada ao trabalho, situando-se entre as primeiras posições no Brasil^1^. Entre as suas principais doenças, estão os distúrbios de voz, doenças do aparelho respiratório e do sistema osteomuscular, além de transtornos mentais e comportamentais^2,3^. Houve um crescimento recente de notificações relacionadas à saúde mental, como no caso do estado de São Paulo^4^. As condições de trabalho a que esses profissionais estão expostos, com longas jornadas, turmas numerosas^5^ e falta de reconhecimento, acabam agravando o problema, com consequências diretas na vida particular dos docentes^6^.

No entanto, ainda há poucos indicadores que analisam as condições dos docentes em cada etapa do ensino básico, desde a educação infantil até o ensino médio, áreas que apresentam distinções e especificidades no contexto de trabalho^7^. Estudos que buscam gerar indicadores normalmente utilizam valores agregados das diferentes etapas do ensino básico^1^ ou são baseados em inquéritos em regiões específicas que dependem da ação do respondente, podendo gerar altos custos operacionais^8,9^.

O uso de indicadores agregados para a análise de doenças pode omitir situações críticas de morbidade e não corresponder à realidade das diferentes condições de trabalho às quais os docentes estão expostos. O problema se agrava ao analisar professores da rede pública municipal, cujos dados de afastamento somente são acessíveis por meio de secretarias de saúde ou departamentos de perícias médicas de cada município^2^. Uma forma de analisar a situação de morbidade por etapa de ensino e a relação do trabalho com o adoecimento de professores é buscar fontes de dados oficiais públicas, que possuam registros individualizados e atualizados com ampla cobertura geográfica.

Nesse cenário, a partir de 2011, o acesso aos dados provenientes do governo federal foi ampliado, mediante a publicação da Lei nº 12.527 de 2011, conhecida como Lei de Acesso à Informação (LAI). Essa legislação permite que sejam solicitadas informações a qualquer órgão público e prevê que as entidades e órgãos públicos se antecipem e publiquem seus dados e informações na internet em formatos abertos e não proprietários, conhecidos como dados abertos^10^. Um exemplo é a Relação Anual de Informações Sociais (RAIS), instituída pelo Decreto nº 76.900, de 23 de dezembro de 1975, atualmente sob responsabilidade do Ministério da Economia. Os dados são divulgados anualmente de forma pública e anônima, contendo registros individualizados com cobertura em todo o território nacional^11^.

A RAIS tem como principal finalidade subsidiar a geração de estatísticas do trabalho e do mercado formal (privado e estatutário) para as entidades governamentais. No entanto, também são disponibilizados os tipos de afastamento, como doença ou doença relacionada ao trabalho, sem informações da doença específica. O preenchimento é obrigatório a todas as empresas públicas e privadas desde 1977^12^. Nos dados de afastamento de professores na RAIS, verifica-se que em média mais de 40% dos professores da educação infantil se afastaram do trabalho ao menos uma vez ao ano devido a morbidades no período entre 2014 e 2018, superando as demais etapas do ensino básico e apontando para a necessidade de analisar esse grupo específico.

O presente estudo propõe construir modelos preditivos capazes de estimar o risco de ausência laboral dos docentes da educação infantil que atuam na rede pública municipal, considerando todos os municípios do estado de São Paulo, utilizando inteligência artificial e algoritmos de aprendizagem supervisionada (*machine learning*) conhecidos pelo alto desempenho, principalmente na área da saúde^13^. Espera-se que o melhor modelo proposto possa ser aplicado para estimar o número de afastamentos por doença em professores, utilizando dados projetados dos trabalhadores (por exemplo, tempo de ocupação, carga horária e renda) e do ambiente de trabalho (por exemplo, alunos por turma), de forma a subsidiar políticas públicas direcionadas de prevenção de ausência laboral, evitando novos adoecimentos, melhorando a qualidade de vida desses profissionais e, consequentemente, a qualidade do ensino na rede pública.

## MÉTODOS

Trata-se de um estudo transversal para predizer risco de afastamento por morbidade (doença ou doença relacionada ao trabalho) em professores que atuam na educação infantil (período pré-escolar) de toda a rede pública municipal do estado de São Paulo, pelo uso de dados secundários, públicos e anônimos.

### Fontes de Dados

Foram utilizados dados individualizados em seu menor nível de desagregação, conhecidos também como microdados^11^, de vínculos empregatícios formais fornecidos pela RAIS no período entre 2014 e 2018. A RAIS mantém informações fornecidas anualmente pelas empresas públicas e privadas sobre os vínculos empregatícios formais de seus funcionários, como contratações e demissões realizadas no ano corrente, além de dados sobre jornada de trabalho e afastamentos por doença ou doença relacionada ao trabalho. Foram incluídos professores da rede pública municipal de todos os municípios do estado de São Paulo que informaram dados à RAIS, nos quais os docentes possuíam nível superior e vínculo empregatício ativo até 31 de dezembro do ano corrente atuando no ensino pré-escolar, classificados na RAIS com a CBO 231105 (Professores de Ensino Superior no Ensino Infantil, quatro a seis anos).

Como forma de identificação do desfecho, foram utilizados os campos de causa de afastamento presentes na RAIS, filtrando-se pelos códigos 30 (doença de trabalho) e 40 (doença). Não foram consideradas aposentadorias decorrentes de doenças. Para a identificação do tipo de estabelecimento (federal, estadual, municipal ou privado), foi utilizada a informação da natureza jurídica do estabelecimento (códigos 1031, 1066, 1120, 1155, 1180 e 1244). Para identificação da atividade do estabelecimento, foram utilizados o código 841160 (administração pública em geral) e a divisão 85 (educação) da Classificação Nacional de Atividades Econômicas versão 2.0 (CNAE 2.0) fornecidos na RAIS.

Cerca de 99,7% (n = 174.346) dos registros estavam cadastrados no CNAE 841160, sendo que o restante, 0,03% (n = 52), pertencia a um CNAE não relacionado ao ensino infantil pré-escolar, sendo excluído da seleção. Também foram excluídos registros em que o município de exercício do trabalhador não correspondia à unidade federativa de São Paulo, resultando no total de 174.294 registros. Nas situações em que o município de exercício do trabalhador não foi informado, foi adotado o município do estabelecimento.

Para capturar parte das condições de trabalho a que esses profissionais estavam expostos, foi vinculada a informação da média de alunos por turma na etapa pré-escolar, fornecida pelo Instituto Nacional de Estudos e Pesquisas Educacionais Anísio Teixeira (Inep)^14^, considerando que algumas doenças comuns aos docentes estão associadas à acústica do ambiente e à complexidade na atuação do docente^3^, e adicionado o número de habitantes por município, fornecido pela Fundação Seade^15^. Os dados do Inep e da Fundação Seade foram vinculados à amostra selecionada da RAIS por meio do código do município no Instituto Brasileiro de Geografia e Estatística (IBGE).

Ao todo, foram selecionadas 11 variáveis preditoras: sexo, idade, pós-graduação, tamanho do estabelecimento, tipo de vínculo empregatício, primeiro emprego, tempo no emprego, horas contratadas, média anual da quantidade de salários mínimos, média de alunos por turma e número de habitantes no município de atuação do trabalhador.

### Preparação dos Dados

Os dados passaram por uma etapa inicial de pré-processamento para tratar as variáveis ausentes e transformá-los para que pudessem ser utilizados na construção dos diferentes tipos de modelos preditivos de *machine learning*^16^. Todas as variáveis selecionadas estavam totalmente preenchidas, sem valores ausentes (*missing*). Variáveis com mais de duas categorias foram representadas por um conjunto de variáveis chamadas *dummy*, em que para cada categoria é gerada uma nova variável com valores de 0 ou 1. As variáveis contínuas foram padronizadas por meio de escore-z (*z-score*). Foi testada a correlação entre as variáveis numéricas e constatada alta correlação entre o número de habitantes no município e a média de alunos (0,93), portanto optou-se por dicotomizar a variável de número de habitantes utilizando a definição de 500 mil habitantes para identificar atuação em cidade grande^17^.

Para a fase de pré-processamento e carga de dados, foi utilizado o sistema gerenciador de banco de dados MS SQL Server. Para a análise de dados e construção dos modelos preditivos, foi utilizado o software R.

### Construção dos Modelos Preditivos

Ao todo, foram desenvolvidos cinco algoritmos de aprendizado supervisionado de *machine learning*: regressão logística, árvores de decisão^16^, *random forest*^18^, *XGBoost*^19^ e redes neurais artificiais^20^. Algoritmos de *machine learning* estão sujeitos a problemas como o subajuste (*underfitting*) – quando o modelo não consegue se ajustar à variabilidade inerente dos dados, gerando estimativas ruins – e sobreajuste (*overfitting*) – quando o modelo se ajusta muito bem para a amostra utilizada no treinamento, mas não obtém bons resultados para novos dados. Uma forma de melhorar o desempenho dos algoritmos e evitar problemas como *overfitting* ou *undefitting* é aplicar técnicas de reamostragem (*resampling*) e validação cruzada (*cross-validation*) para a seleção de hiperparâmetros^16^.

Na validação cruzada, o conjunto de dados é separado em duas partes. A primeira parte é destinada ao treinamento do algoritmo e a segunda parte é utilizada para ajustes dos hiperparâmetros do modelo, simulando novos dados e selecionando hiperparâmetros que otimizem a métrica de performance escolhida. Essa fase de treinamento permite que o algoritmo obtenha um melhor desempenho preditivo quando são apresentados novos conjuntos de dados. Uma das técnicas de validação cruzada mais conhecidas é a *k-fold,* na qual *k* significa o número de subconjuntos dos dados de testes que serão utilizados para ajustes dos hiperparâmetros do modelo durante a fase de treinamento^21^.

Para a construção dos modelos, separou-se uma parte dos dados (60%) referente ao período entre 2014 e 2016 (n = 103.357) para treinamento, incluindo validação cruzada com a técnica *k-fold* utilizando 10 partições para definição dos hiperparâmetros. A outra parte dos registros (40%), referente ao período entre 2017 e 2018 (n = 70.937), foi utilizada para a avaliação de desempenho dos modelos, omitindo-se o resultado sobre afastamento por doença e aplicando o modelo treinado para estimar o desfecho em dados futuros. Para avaliar o desempenho dos modelos, foram analisadas medidas como acurácia, sensibilidade, especificidade, valor preditivo positivo (PPV) e valor preditivo negativo (NPV). Para selecionar o melhor modelo, foi utilizado o valor da área abaixo da curva *receiver operating characteristic* (AUROC)^22^. Foi utilizado o coeficiente kappa de Cohen para avaliar a concordância entre os valores previstos e observados.

Este estudo segue as orientações de descrição de modelos preditivos multivariados para diagnóstico ou prognóstico (*Transparent Reporting of a Multivariable Prediction Model for Individual Prognosis or Diagnosis* – TRIPOD)^23^.

### Critérios de Confidencialidade

O projeto foi aprovado pelo Comitê de Ética em Pesquisa da Faculdade de Saúde Pública da Universidade de São Paulo, sob o nº 4.031.362, CAAE 30786620.5.0000.5421.

## RESULTADOS

### População de Estudo

Foi realizada inicialmente a análise descritiva dos dados de treinamento (2014 a 2016) e teste (2017 a 2018). Os dados de treinamento compreendem 103.357 registros de vínculos empregatícios de professores, havendo 45.419 (43,94%) registros de afastamentos por doença ou doença relacionada ao trabalho. Os dados de teste correspondem a 70.937 registros e 30.261 casos de afastamento por doença (42,65%). A Tabela 1 mostra os resultados dessa análise.

**Tabela 1:** Análise descritiva dos dados de treinamento (n = 103.357) e teste (n = 70.937).

Variável	Categoria	Treinamento		Teste	
		n	%	n	%
Sexo	0 – Masculino	22.120	21,40	16.055	22,63
	1 – Feminino	81.237	78,60	54.882	77,37
Pós-graduação	0 – Não	100.904	97,63	68.412	96,44
	1 – Sim	2.453	2,37	2.525	3,56
Idade^a^	-	43,74 (9,73)		43,97 (9,70)	
Empresa grande^b^	0 – Não	19	0,02	19	0,03
	1 – Sim	103.338	99,98	70.918	99,97
Tipo de vínculo	0 – Estatutário	96.682	93,54	65.210	91,93
	1 – CLT	5.867	5,68	4.493	6,33
	2 – Temporário	808	0,78	1.234	1,74
Primeiro emprego	0 – Não	100.105	96,85	65.236	91,96
	1 – Sim	3.252	3,15	5.701	8,04
Tempo no emprego (meses)^a^	-	120,49 (92,31)		123,29 (93,84)	
Horas contratadas^a^	-	33,28 (6,60)		33,28 (6,33)	
Salários mínimos^a^	-	5,03 (3,04)		5,04 (3,04)	
Cidade grande^c^	0 – Até 500 mil hab.	30.866	29,86	22.212	31,31
	1 – Acima de 500 mil hab.	72.491	70,14	48.725	68,69
Alunos por turma^a^	-	27,42 (5,75)		26,74 (5,50)	

hab.: habitantes

^a^ Média (desvio-padrão).

^b^ Acima de 100 funcionários.

^c^ Classificação de cidades pela Organização para a Cooperação e Desenvolvimento Econômico (OCDE)^17^: pequena, média, grande (acima de 500 mil habitantes) e metrópole.

Em seguida, foi realizada a análise das variáveis independentes para verificar se há relação significativa com o desfecho, utilizando o teste de associação pelo qui-quadrado de Pearson para as variáveis categóricas. A Tabela 2 mostra os resultados e permite observar que a maioria das variáveis apresenta associação significativa com o desfecho.

**Tabela 2:** Análise de associação com o desfecho da amostra de treinamento.

Variável	Categoria	Professores afastados por doença			Teste c^2a^
		Sim	Não	Total	
		n (%)	n (%)	n (%)	
Dados do trabalhador					
Sexo	0 – Masculino	9.930 (44,89)	12.190 (55,11)	22.120 (100,0)	< 0,001
	1 – Feminino	35.489 (43,69)	45.748 (56,31)	81.237 (100,0)	
Pós-graduação	0 – Não	45.075 (44,67)	55.829 (55,33)	100.904 (100,0)	< 0,0001
	1 – Sim	344 (14,02)	2.109 (85,98)	2.453 (100,0)	
Idade	43,74 (9,73)				
Dados da empresa					
Empresa grande^b^	0 – Não	11 (57,89)	8 (42,11)	19 (100,00)	0,220
	1 – Sim	45.408 (43,94)	57.930 (56,06)	103.338 (100,00)	
Dados do vínculo					
Tipo de vínculo	0 – CLT	746 (12,72)	5.121 (87,28)	5.867 (100,0)	< 0,0001
	1 – Estatutário	44,500 (46,03)	52.182 (53,97)	96.682 (100,0)	
	2 – Temporário	173 (21,41)	635 (78,59)	808 (100,0)	
Primeiro emprego	0 – Não	44.616 (44,57)	55.489 (55,43)	100.105 (100,0)	< 0,0001
	1 – Sim	803 (24,69)	2.449 (75,31)	3.252 (100,0)	
Tempo no emprego (meses)	120,49 (92,31)				
Horas contratadas	33,28 (6,60)				
Salários mínimos	5,03 (3,04)				
Dados ambientais					
Cidade grande^c^	0 – Até 500 mil hab.	3.422 (11,09)	27.444 (88,91)	30.866 (100,0)	< 0,0001
	1 – Acima de 500 mil hab.	41.997 (57,93)	30.494 (42,07)	72.491 (100,0)	
Alunos por turma	27,04 (5,60)				

hab.: habitantes

^a^ Teste de associação pelo c^2^: *H*_0_: não existe associação; H_a_: existe associação.

^b^ Acima de 100 funcionários.

^c^ Classificação de cidades pela Organização para a Cooperação e Desenvolvimento Econômico (OCDE)^17^: pequena, média, grande (acima de 500 mil habitantes) e metrópole.

Nota: Valores em média (desvio-padrão).

### Análise dos Modelos Preditivos

A Figura 1 mostra as curvas ROC de cada modelo desenvolvido, nos quais foram incluídas 11 variáveis (tipo de vínculo empregatício, número de horas contratadas, tempo de emprego em meses, média da quantidade de salários mínimos do ano, sexo, tamanho do estabelecimento, possuir pós-graduação, idade, primeiro emprego, média de alunos por turma pré-escolar no município e número de habitantes).

**Figura 1:** Comparação dos modelos com base nos resultados de predição utilizando dados de teste (2017 e 2018).

A Tabela 3 mostra os resultados do desempenho de cada algoritmo. O melhor modelo foi o de redes neurais artificiais, que obteve 0,79 de área abaixo da curva, com acurácia de 71,52%, sensibilidade de 72,86%, especificidade de 70,52%, PPV de 64,77% e NPV de 77,74%. O modelo também apresentou a melhor coeficiente kappa, de 0,4266.

**Tabela 3:** Resultados dos modelos desenvolvidos utilizando dados de teste.

Algoritmo	AUC	Sensibilidade	Especificidade	Acurácia	PPV	NPV	Kappa
Regressão logística	0,7792 [0,7759–0,7826]	0,7754	0,6568	0,7074	0,6270	0,7972	0,4195
Árvore de decisão	0,7756 [0,7722–0,7790]	0,7008	0,7243	0,7142	0,6541	0,7649	0,4212
*Random forest*	0,7670 [0,7634–0,7703]	0,7715	0,6571	0,7059	0,6260	0,7944	0,4134
*XGBoost*	0,7843 [0,7810–0,7876]	0,7235	0,6970	0,7083	0,6398	0,6970	0,4136
Redes neurais artificiais	0,7902 [0,7867–0,7934]	0,7286	0,7052	0,7152	0,6477	0,7774	0,4266

AUC: área abaixo da curva

Os hiperparâmetros dos modelos de *machine learning* que obtiveram os maiores valores de AUC foram os seguintes: árvore de decisão (cp-complexity-parameter = 0.000385), *random forest* (mtry = 7), *XGBoost* (nrounds = 100, max_depth = 4, gamma = 0.6, colsample_bytree = 0.8, min_child_weight = 2, subsample = 1) e redes neurais artificiais (size = 19, decay = 0.2).

Cada algoritmo utiliza um conjunto de regras diferentes para se ajustar aos dados com o menor erro possível, sendo que algumas variáveis se tornam mais importantes que outras para a capacidade preditiva do algoritmo. A importância das variáveis refere-se a quanto a variável contribuiu para realizar predições acuradas em cada modelo. Para os modelos lineares, utiliza-se de estatística t tradicional para contabilizar a importância das variáveis. Nos demais modelos, a importância depende da implementação de cada algoritmo.

Por exemplo, no caso de árvores de decisão, se uma variável é utilizada com maior frequência nas divisões de folhas contribuindo para maior pureza do nó, ela terá uma importância maior para o modelo final, significando que sua remoção prejudicará a performance geral do modelo. Nos modelos que utilizam múltiplas árvores, como o *random forest*, a importância das variáveis é calculada a cada divisão das folhas de uma árvore e somada com o resultado das demais árvores^21^. Nos modelos de redes neurais, para este estudo foi utilizado o método “*weights*”^24^, em que se utiliza uma combinação dos valores de saída dos neurônios das camadas ocultas e os valores dos pesos de cada conexão para calcular a maior contribuição e representatividade de cada variável de entrada no resultado da classificação. Neste estudo, foi utilizado o pacote caret^25^ do R, que já implementa o cálculo de importância de variáveis de acordo com cada modelo^26^.

Apesar de não ter interpretação causal^27^, as variáveis podem dar informações relevantes sobre a sua relação com o desfecho. A Figura 2 demonstra como cada variável contribuiu para os modelos desenvolvidos.

**Figura 2:** Importância das variáveis por modelo preditivo.

As variáveis que mais contribuíram para o melhor modelo (redes neurais artificiais) foram: quantidade de salários mínimos, cidade grande, média de alunos por turma, tempo no emprego e empresa grande.

## DISCUSSÃO

A RAIS pode fornecer indícios da situação de morbidade dos trabalhadores por meio do campo de causa de afastamento. A ampla cobertura e o registro individualizado facilitam a seleção e análise de dados de uma população de trabalhadores formais com a mesma ocupação em um determinado estado, o que permite acompanhar ao longo dos anos a evolução de afastamentos por doença e predizer quais profissionais apresentam maior risco de adoecimento nos próximos anos. Para o melhor algoritmo (redes neurais artificiais), obteve-se 0,79 da área abaixo da curva ROC, sensibilidade de 72,86% e 71,52% de acurácia, usando apenas 11 variáveis preditoras.

Os resultados mostram a viabilidade da utilização de fontes anonimizadas para a predição de morbidade de docentes do ensino pré-escolar com algoritmos de *machine learning*. As variáveis que mais contribuíram para o melhor modelo podem ser exploradas em estudos futuros, para verificar se há relação causal. Porte da cidade em que se trabalha, renda média do trabalhador e média de alunos por turma podem ter impactos diretos na saúde dos professores. No caso da média de alunos, há evidências na literatura que corroboram este raciocínio, como problemas na acústica do ambiente e elevado ruído devido a maior agitação em sala de aula^3,28^.

Algoritmos de *machine learning* têm sido utilizados em diferentes contextos na saúde do trabalhador para auxiliar na predição de morbidades relacionadas às atividades destes profissionais^29,30^. Na área da educação, não foram encontrados estudos que utilizaram *machine learning* ou demais modelos preditivos para risco de absenteísmo docente como principal desfecho, havendo alguns estudos que analisaram a associação de variáveis preditoras com o desfecho^31,32^, não sendo sua predição o principal objetivo. A predição de absenteísmo docente pode identificar professores que precisam de maior atenção, permitindo adotar medidas para evitar afastamentos por doenças relacionadas à ocupação^3,28^, diminuindo a ausência ao trabalho e consequentemente aprimorando a qualidade do ensino^33^.

O estudo possui algumas limitações. Por se tratar de dados anônimos, não foi possível verificar se os docentes, que podem ter múltiplos vínculos empregatícios municipais, atuam em mais de uma escola. Também não foi possível identificar se houve um afastamento prévio por doença pré-existente que possa estar relacionado aos afastamentos seguintes. Por fim, foi analisado um número baixo de variáveis preditoras devido à disponibilidade da RAIS. Espera-se que no futuro, com uma coleta mais extensa realizada por meio de questionários, sejam possíveis resultados ainda mais robustos por parte dos algoritmos.

Em conclusão, somente com dados públicos e anônimos, foi possível obter boa sensibilidade (73%) e especificidade (71%) na identificação de risco de absenteísmo por doença, independentemente da causa da morbidade. Apesar de não ter sido criada para fins da saúde, a RAIS mostrou-se viável para análise de absenteísmo por morbidades, principalmente por conter variáveis das relações de trabalho, podendo ser explorada em outras categorias profissionais. Os modelos desenvolvidos podem futuramente ser aplicados nas demais etapas de ensino e em outras categorias ocupacionais. Algoritmos de *machine learning* podem contribuir com predições mais assertivas na análise de absenteísmo de professores do ensino infantil da rede pública municipal, podendo ser usados na elaboração de indicadores de morbidade ou para subsidiar políticas públicas prevencionistas direcionadas a essa categoria profissional.
